# Just a Cog in the Machine? The Individual Responsibility of Researchers in Nanotechnology is a Duty to Collectivize

**DOI:** 10.1007/s11948-015-9718-1

**Published:** 2015-11-04

**Authors:** Shannon L. Spruit, Gordon D. Hoople, David A. Rolfe

**Affiliations:** Department of Values, Technology and Innovation, Delft University of Technology, P.O. Box 5015, 2600 GA Delft, The Netherlands; Department of Mechanical Engineering, University of California, Berkeley, Berkeley, CA USA; Materials Science and Corrosion Practice, Exponent Inc., Menlo Park, CA USA

**Keywords:** Responsible Research and Innovation, Collectivization duty, Interdisciplinary, Nanotechnology, Emerging technologies, Collective responsibility

## Abstract

Responsible Research and Innovation (RRI) provides a framework for judging the ethical qualities of innovation processes, however guidance for researchers on how to implement such practices is limited. Exploring RRI in the context of nanotechnology, this paper examines how the dispersed and interdisciplinary nature of the nanotechnology field somewhat hampers the abilities of individual researchers to control the innovation process. The ad-hoc nature of the field of nanotechnology, with its fluid boundaries and elusive membership, has thus far failed to establish a strong collective agent, such as a professional organization, through which researchers could collectively steer technological development in light of social and environmental needs. In this case, individual researchers cannot innovate responsibly purely by themselves, but there is also no structural framework to ensure that responsible development of nanotechnologies takes place. We argue that, in such a case, individual researchers have a duty to collectivize. In short, researchers in situations where it is challenging for individual agents to achieve the goals of RRI are compelled to develop organizations to facilitate RRI. In this paper we establish and discuss the criteria under which individual researchers have this duty to collectivize.

## Introduction

Responsible Research and Innovation (RRI) is emerging as one of the leading paradigms for discussions concerning the governance of new technologies. Broadly speaking, research and innovation following the RRI praxis should lead to the development of technologies that promote social and environmental values (Owen et al. 2013; Van den Hoven 2013), and respond to the grand challenges of our time (von Schomberg 2013). While few engineers and scientists would disagree with the goals of incorporating social and environmental values into research and innovation, how to accomplish such lofty goals is not so clear. Consider technological innovations that are characterized by a high degree of uncertainty. In the early stages of development it is difficult to predict the impacts of the technology, whereas in later stages the ramifications may be more difficult to rectify due to entrenchment and technological lock-in (Collingridge 1981). Scholars involved in the discussion concerning RRI aspire to provide an answer to this ‘dilemma of control’ by developing RRI as a framework that is both flexible and responsive, in order to be able to adapt to new (scientific) information and changing ethical perspectives on technological impacts (Alfred Nordmann 2014; Owen et al. 2013).

RRI is characterized by a shift from assessing the desirability of the *outcome* of innovation processes, such as evaluating harmful product outcomes in court under liability law, to assessing the qualities of the innovation *proce*ss. Drawing heavily on ideas like constructive technology assessment (Rip and Te Kulve 2008), midstream modulation (Fisher et al. 2006; Schuurbiers 2011) and anticipatory governance (Guston 2013; Sarewitz 2011), several authors have proposed methodologies for assessing the responsibility of research and innovation. The RRI paradigm proposed by Stilgoe, Owen, Macnagten and their colleagues has four dimensions (Owen et al. 2013; Stilgoe et al. 2013). They propose that research should be: anticipatory, exploring in advance and anticipating potential technological impacts; reflective, by examining goals and purposes of technologies as well as the uncertainties in risk assessment; deliberative, the idea that public and diverse stakeholders’ perspectives are actively considered during design processes and, lastly; responsive, the actual alteration and shaping of technological trajectories in response to deliberation and reflection. van den Hoven (2013, 2009) proposes Value Sensitive Design as a methodology for RRI; conceptual and empirical research with the aim of value identification, and a value-directed design methodology would be needed in order to make innovation more responsible. Van de Poel writes about the responsibility of innovation processes in terms of responsible experimentation (2009, 2011). Since research and innovation in new and emerging technological fields are prone to uncertainty, we should perceive them as forms of social experimentation. Van de Poel argues that such experiments should be governed by normative requirements drawn from the realm of bioethics and medical experimentation.

The process orientation that is integral to RRI is predicated on some form of organization. The abovementioned approaches all assume that technological development trajectories are actively steered in light of new information perspectives on technological risks, or other socially or environmentally undesirable impacts. This is a core tenet of RRI. It follows that we must then ask: who is actually doing RRI? Processes cannot be responsible, nor can they reflect on or account for what they do and make intentional choices. In the end, responsibility should rest with a particular agent. RRI refers to a collection of individual agents that perceive, reflect, and act together in such a way that this leads to technologies being designed with certain values in mind. While it may make sense to set process level requirements when designing a governance structure, when it comes down to allocating responsibility we must focus on individual agents since individuals, not processes, can be the subject of responsibility claims. They are the ones who, under the header of RRI, should “either feel responsible, or can be held or can be made responsible” for the course of innovation processes (Van den Hoven 2013, 81).

The purpose of this paper is to elaborate the responsibilities of individual researchers in RRI. We argue that, for engineers and scientists to successfully implement RRI, they have a *duty to collectivize* and must develop organizations to facilitate RRI since this is so difficult for individual agents to achieve on their own. We use nanotechnology as a case study to develop our argument, in part because the National Nanotechnology Initiative in the United States has identified supporting the responsible development of nanotechnology as one of its four primary goals. We focus on the nanotechnology field at the University of California, Berkeley (UC Berkeley), a world leader in nanotechnological research. We first explore how the emerging and enabling nature of nanotechnology makes it difficult for individual researchers to contribute to RRI. We examine how the limited individual capacity of researchers to control and foresee nanotechnological development impedes the allocation of responsibility. Next, we argue that there is no collective agent within the nanotechnology field that could be capable of RRI. Then, we turn to a more theoretical discussion on how the dispersed nature of the nanotechnology field impacts individual responsibility. We develop a set of criteria to argue that researchers involved in nanotechnology have a duty to collectivize, to organize themselves into a collective that can innovate responsibility. This framework offers a new perspective on individual responsibility for RRI.

## Research Approach

This paper is the product of a collaborative study, or rather a collaborative reflection, on the ethical dimensions of nanotechnologies. From the summer of 2013 to the spring of 2015, a group consisting of two PhD students in engineering from the UC Berkeley and one PhD student in ethics from Delft University of Technology participated in the jointly organized program ‘Global Perspectives: Engineering Ethics Across International and Academic Borders’ (Sunderland et al. 2014). During this program, the authors were sensitized to each other’s work and encouraged to come up with joint research projects, using collaboration as a form of research (Calvert 2014).

Nanotechnologies are a central theme in the work of all three the authors, though GH and DR are involved in it from an engineering perspective and SS from an ethical perspective. We easily engaged in discussions concerning ethical issues in engineering work; however how to operationalize ethics in nanoscience and technology was not at all obvious. For instance, after reading nanoethics texts for the Global Perspectives program, GH and DR felt the field was often too speculative and inclusive of non-nanotechnologies. We all experienced the field as ambiguously defined and found its terminology heavily skewed by the external pressures of funding and networking activities (Nordmann 2007). Furthermore, little work seemed to address DR’s immediate concerns about nanotechnologies, such as the use and risks of nanomaterials in working environments and the effect of nanoparticles on the environment. At the other end of the spectrum, SS was surprised by how little room was reserved for discussions of such topics within daily research practices at UC Berkeley. Along the way we realized that, for engineers and ethicists to find each other’s work relevant, it is critical to develop a shared understanding of what the field of nanotechnology entails as well as what responsibilities come with being a member of the nanotechnology community. This paper is a first attempt to develop such a shared understanding, and can be read as a self-reflection and a result of the position that we take within the field: PhD researchers.

The empirical sections of this paper are based on the local contexts of UC Berkeley, and describe the academic setting experienced by GH and DR. UC Berkeley is an academic leader in nanotechnology with over 100 faculty across ten departments researching in this area. That said, we think our analysis is generally representative; we take it that the dispersed structure around nanotechnology research is fairly representative of the way nanoengineering research is conducted at top research institutions in the United States and sheds light on the more general topic of how converging fields with no clear and monolithic institutional space (such as a stand-alone department) impede individual responsibility-taking.

## Responsible Research and Innovation in Nanotechnology

In 2000, Bill Clinton launched the National Nanotechnology Initiative (NNI), a research program devoted to advancing understanding and control of matter at the nanoscale (Sargent 2014). Since then, the United States federal government has invested $19.4 billion in the program. The initiative has led to a wide range of innovations, ranging from breakthroughs in battery technology to nanoscale transistors. Nanotechnology may be thought of as a range of tools that enable advancements in other fields, including biology, electrical engineering, materials science, and physics. Nanotechnologies are of great interest to scientists and engineers because the physical nature of matter and energy changes as we reach the nanoscale. When matter is constrained to the scale of nanometers in at least one dimension, roughly 10^−9^ m, it exhibits novel physical, electrical, and optical properties. The most notable of these changes is the presence of quantum effects that cannot be seen in larger materials. These properties allow scientists and engineers to create improvements across disciplines such as surface interactions, molecular biology, semiconductor physics, and microfabrication. Nanotechnology research extends from fundamental science to consumer applications, which makes it difficult to develop an overall approach for enabling nanotechnological researchers to evaluate and address the ethical risks posed by their work. This is particularly true for researchers of fundamental nanoscale physics and cutting-edge nanofabrication technologies. Research in these fields seeks to understand the physical phenomena and fabrication techniques that may later serve as tools for a wide range of disciplines. These approaches are often implemented into applications that the initial researcher had never imagined.

One of the four stated goals of the NNI program is to “support responsible development of nanotechnology” (National Science and Technology Council 2014; 34). While this seems to suggest that nanotechnology is a uniform and distinct field, posing a unique set of ethical concerns, in practice, nanotechnological research is interdisciplinary, dispersed, with many actors and structures governing the funding of and research in this field. Nanotechnology research has advanced widely, but only a single major research institution (UC San Diego) has created a stand-alone nanotechnology department. Elsewhere, researchers are spread across many departments based on the applications of their nanotechnological research. So most nanotechnological research exists within an application discipline. This structural arrangement facilitates collaboration between experts in nanotechnology and experts in application fields. Interactions between actors within nanotechnology are limited to more informal structures such as shared research spaces, funding initiatives, seminar groups, and certificate programs. In practice, this limits the forums for nanotechnological researchers to discuss ethical responsibilities that may be unique to nanotechnology. Thus the key question is: given the state of the nanotechnology community, how can individual researchers achieve the NNI’s goal of supporting responsible development of nanotechnology?

### Limitations on the Capacity of Individual Researchers to Steer Nanotechnological Development

By working with materials and devices at the nanoscale, scientists and engineers are able to make significant advances in a wide range of industries, including drug delivery, transportation, weaponry, and microprocessors (Lin and Allhof 2007). Here we argue that this structure makes it challenging for individual researchers to steer nanotechnological development, because it impedes their capacity to control and foresee how nanotechnologies will be developed and applied.

A key characteristic of nanoscience and nanotechnologies is that they play an enabling role in other fields. The enabling effects of nanotechnology can be broken down into two basic categories: sustaining innovations and disruptive innovations. Computer processors are an excellent example of how nanotechnology enables sustaining innovation. In 1965, Gordon Moore, co-founder of Intel, predicted that the number of transistors on a computer processor would double every 2 years. Maintaining this pace over the last five decades has forced engineers to constantly find ways to reduce the size of transistors. Without the enabling effects of nanotechnology, Moore’s law would have failed long ago. While the process for making computer components has not fundamentally changed, the ability to manufacture at smaller and smaller sizes has made new chipsets possible. In other fields nanotechnology has acted as a disruptive innovation, enabling previously inconceivable discoveries. Biological systems are inherently nanostructured and nanofunctional, so the advent of nanotechnology allows for a literal quantum leap in medical systems. Some of the most promising early applications of nanomedicine have come in the realm of drug delivery. Polymer-drug nanoparticles and nanodrug delivery devices with variable diffusivity allow drugs to be delivered to targeted regions of the body (LaVan et al. 2003). In applications such as anti-cancer drugs, such technology could target tumors while protecting healthy cells from toxic exposure. Nanotechnology has also allowed for the synthesis of tissues that might 1 day become implantable organs (Griffith and Naughton 2002).

A consequence of developing an enabling technology is that inventors must relinquish control of their inventions to those who use the technology. The emergence of nanoscience and nanotechnology has created, and continues to create, opportunities for research and technological development beyond the scope of the nanotechnology community. The capacity of individual scientists and engineers to steer or adjust nanotechnological developments is strongly determined by their influence on the fields in which their technologies are going to be applied. Although most nanotechnological research occurs in a university setting, the resulting ideas are commercialized by corporate entities that license the intellectual property. In such cases the capacity of the basic researcher to influence nanotechnological applications could be even more limited.

Furthermore, as researchers in nanotechnology do groundbreaking work they may be unwittingly laying the scientific framework on which applications will later be based. In a way these nanotechnologies are like hammers looking for nails; their effects only materialize because of their enabling effects. Researchers may have a particular application in mind during the development phase, but often it is the unexpected or unanticipated discovery that produces the most interesting technology. Consider the case of quantum dots. Energy level confinement was first discovered in 1974, with the name ‘quantum dot’ first applied in 1988 (Reed et al. 1988). Forty years later, the initial discovery has led to transistors, solar cells, LEDs, and diode lasers. In 2013, a flat-panel television became the first example of a commercial technology incorporating quantum dots (Bullis 2013). Nearly 40 years passed before the initial innovation resulted in a commercial application.

Consequently, it seems unreasonable to expect individual researchers to bear the responsibility and to be able to steer nanotechnological trajectories resulting from their enabling work. Scientists and engineers working on nanotechnology are only able to oversee a relatively small part of the innovation process. This is, of course, not a new critique; a similar argument has been made by Swierstra and Jelsma who render individual accountability for technological development problematic because of the collaborative nature of science (Swierstra and Jelsma 2006). Parallels can also be drawn with discussions of ‘many hands problems’ in engineering contexts, in which a group of actors cause harm through their combined behaviors rather than individual wrongdoing. In such cases the distribution of labor also means that individuals lack the capacity to prevent major harm single-handedly.

While the challenges of steering technological trajectories are indisputable, does this absolve enabling scientists and engineers of all moral responsibility for how their technology is used? We think not, since nanotechnology researchers have still contributed to the innovation processes, even though their individual impact takes place over large spans of time. In a sense, individual researchers share a responsibility with all those involved in nanotechnological developments. While they may not be individually responsible, by contributing to a shared innovation trajectory they seem to play a part in a larger collective that may be a reasonable candidate for bearing this forward-looking responsibility (Miller 2006). Next, we will explore the extent to which the nanotechnology community as a whole could serve as a vehicle for allocating forward-looking responsibility to individuals.

### The Problem with Defining a Collective Agent in the Nanotechnology Field

We have concluded that, if we want nanotechnology to be developed responsibly, individual researchers must in some sense share this responsibility with others involved in the nanotechnology research and development process. Because of their higher level of organization, such groups of people may constitute a collective that can bear forward-looking responsibility. Collective agents are generally distinguished from mere aggregates of people acting in parallel by (1) the existence of some sort of group decision mechanism, (2) the achievement of, or aspiration to achieve, certain common aims, and (3) the assignment of roles and tasks to group members in order to achieve those aims (Collins 2012; Pettit and Schweikard 2006). Given the fact that individual researchers have a limited capacity to act, this section will explore the extent to which the field of nanotechnology constitutes a collective or group agent structure with the capacity to steer nanotechnological development.

Nanotechnology is a highly interdisciplinary field; scientists in many fields have sought and found ways to understand, manipulate and create matter at the nanometer scale. As many nanotechnologies are broadly applicable, one of the other ways nanoscientists and nanoengineers interact is around particular research thrusts. Consider again the example of quantum dots. These dots are a topic of study for many researchers at UC Berkeley, including physicists, chemists, materials scientists, and engineers. Among them are Stephen R. Leone, Professor of Chemistry and Physics, and Paul Alivisatos, Professor of Chemistry and Materials Science & Engineering, who have been cross-appointed to a total of four different departments, illustrating how their research transcends traditional disciplines. Professor Leone’s research page lists a wide array of topics relating to physics and chemistry, including “ultrafast laser investigations and soft x-ray probing of valence and core levels” and “nanoparticle fluorescence intermittency” (Department of Chemistry 2014a, b). Professor Alivisatos’ biography states that his “research concerns the structural, thermodynamic, optical, and electrical properties of colloidal inorganic nanocrystals” (Department of Chemistry 2014a). Despite different research thrusts, both professors are interested in quantum dots. They have independently published several papers concerning quantum dots and have also collaborated on one publication (Vura-Weis et al. 2013).

**Fig. 1:**
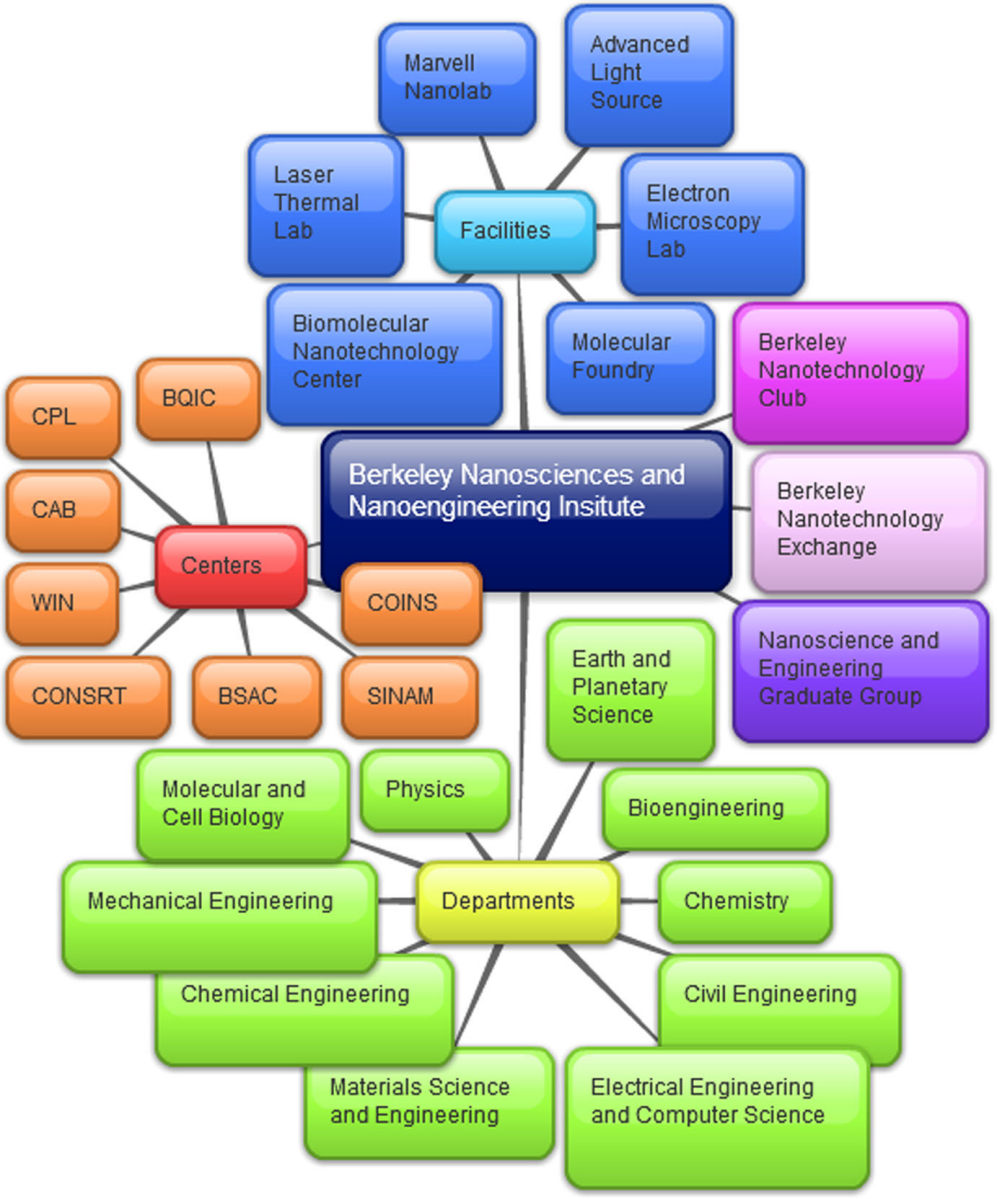
Overview of Nanoscience and Nanotechnology at University of California Berkeley. On top you can find the shared facilities that fall under the header of nanotechnology (*on top*) such as the Marvell Nanolab discussed in the text. The academic departments involved in the Berkeley Nanosciences and Nanoengineering institute are listed at the bottom. On the left several specialized research centers are listed (in counter-clockwise order); The Berkeley Quantum Information and Computation Center (BQIC), The Cell Propulsion Lab (CPL), Center for Analytical Biotechnology (CAB), Western Institute of Nanoelectronics (WIN), a DARPAfunded research center in nano-opto-electronics (CONSRT), Berkeley Sensor and Actuator Center (BSAC), the Center for Scalable and Integrated Nanomanufacturing (SINAM) and Center of Integrated Nanomechanical Systems (COINS). On the right handside you can find The Nanotechnology club, which organizes events for graduate students and undergraduates to educated them about nanotechnology. The graduate nanotechnology group directs the special emphasis in nanotechnology that is part of doctoral education, and is responsible for crosslisting classes in the Department of Nanoscience and Engineering (which has no professors, labs or majors). The nanotechnology exchange is an industrial outreach program, that lets corporate sponsors find research and labs that they are interested in

While these two researchers and their research groups are certainly part of a community, they are housed in different departments and pursue different research goals. They do not seem to meet the definition of a collective agent. Unlike computer science or electrical engineering, nanoengineering is not its own discipline, with a common sense of direction, such as the build up of a shared methodology and body of knowledge. At UC Berkeley the footprint of the nanotechnology collaboration is literally spread over the entire campus. There are over 100 faculty working in nanoscience and nanotechnology, with a multitude of different research thrusts, centers, and collaborations. While describing the full network of actors is beyond the scope of this paper, Fig. 1 gives an overview of the complexity of the network.

In terms of distributing roles or providing a shared decision-making procedure needed for collective agency, the community of scientists and engineers involved in nanoscience and engineering is not a strong organizing force. Researchers involved in nanotechnological development are not part of an institutional framework comparable to those offered by professional organizations in other engineering fields. The UC Berkeley-based Berkeley Nanoscience and Nanotechnology Initiatives (BNNI) aims to be “the umbrella organization for expanding and coordinating Berkeley research and educational activities in nanoscale science and engineering” (BNNI 2007; see Fig. 1). The organization’s website lists researchers working on topics ranging from nanomanufacturing, quantum information and computers to analytical biotechnology. While the BNNI provides a framework for collaboration at UC Berkeley, participation in the nanotechnology community is largely voluntary. Faculty members belong to a home department, such as materials science, but do not share a common nanoengineering department and may choose to participate in some or none of BNNI’s activities. In practice, the type of informal interaction between Leone and Alivisatos discussed above is exemplary for the nanotechnology community at UC Berkeley.^1^

Although nanotechnology researchers are unified by the fact that they have all found different ways to understand, manipulate, and/or create matter at the nanometer scale, this does not mean that they can be regarded as one collective or one professional group. The ad-hoc nature of the field of nanoscience and nanotechnology, with its fluid boundaries and elusive membership, does not establish a collective agent through which scientists and engineers could steer technological development together in light of any unwanted outcomes of nanotechnological innovation.^2^ This causes a paradoxical situation, in which individual researchers cannot innovate responsibly purely by themselves, while there is also no structural framework to ensure that responsible development of nanotechnologies takes place.

## Reframing the Responsibility of Nanotechnology Researchers as a Duty to Collectivize

The nanotechnology field clearly poses a problem for RRI. While RRI requires some sort of synchronized action, this is challenging due to the dispersed nature of the field. It has been convincingly argued, however, that this does not provide sufficient reason for scientists and engineers to avoid or be excused from responsibility (Davis 2012). Nanotechnology researchers are not just a cog in the machine; they are integral contributors to the invention of nanotechnologies. This section explores a solution to this paradoxical situation, by arguing that these researchers may have a *duty to collectivize*.

Unorganized groups of people pose a problem for allocating responsibility. In the previous section we saw that the level of organization and distribution of roles, the presence of a shared decision-making structure, and shared aims all play a role in determining whether or not we can consider a group a collective agent. We do not presuppose a holistic definition of a group agent. In our view a group is basically an organized set of individuals. Our goal is not to focus on the ontological underpinnings of collective agents. Instead we depart from a fairly uncontroversial assumption that a group’s level of coherence and structure influences the extent to which that group of people can be expected, within reason, to be responsible for the behaviors of other group members. Even if no clear group agent exists, we may have the intuition that some sort of shared responsibility is in place. Held (1991) argues that groups that lack the structural organization needed in order to be considered an agent can sometimes be held accountable for collective omissions. For instance, consider strangers on the beach who are confronted with somebody drowning. These strangers have a certain obligation to work together to save the individual from drowning. In such cases we may not hold individuals accountable for the drowning person, but, as Held argues, we would blame these groups of bystanders for not teaming up to save the drowning person. This implies that a random collection of bystanders may perhaps not be a collective agent in the strict sense (having a shared decision-making structure or structural organization), but as group of individuals they have some sort of collective capacity to act.

Collins (2012) builds on Held’s idea and proposes a more formalized account of the intuition that, in some cases, individuals have a responsibility to create a collective agent. In short, Collins argues that, in cases where there is a morally pressing issue that could be resolved by a group, but no group agent exists to resolve it, individual agents may have a duty to create a group, a collective agent, that is capable of resolving the issue concerned (Collins 2012). For this duty to collectivize to apply, Collins suggests the following Criteria for Collectivization Duties (CCD), which we will discuss and apply to the context of nanotechnology research (CCD-Nano).

### Criteria for Collectivization Duties

Collins starts off with five conditions that describe the situations in which collectivization duties are invoked. The first condition defines the moral problem at hand: “ϕ is morally pressing” (Collins 2012, 244), which means that there is an activity ϕ, that must be performed because it is morally valuable in itself or because it brings about a certain morally desirable outcome. In the debates concerning RRI we can recognize both meanings of morally pressing: the incorporation of multiple values into science and engineering is seen as a democratic goal in itself, while it would also lead to the development of technologies that are socially and environmentally acceptable. This then leads to a reformulation of this criterion that overlaps with the stated goal of the NNI: Responsible Research and Innovation of nanotechnology is morally pressing.

The next condition declares that there is nobody, either an individual person or a collective, who actually has a duty to perform the activity ϕ (in our case: responsible development) that would bring about desirable outcomes: “at t_1_, either: no (collective or individual) agent/s have duties, either to ϕ or to take responsive actions with a view to there being the morally desirable outcome that ϕ produces; or too many agent/s with such duties default” (Collins 2012, 244). Two kinds of duties are mentioned in this condition. The first is a direct duty to ϕ and the second is a duty to take responsive actions to ϕ, thus a duty to contribute to ϕ taking place. We interpreted the first direct duty as the duty of RRI, for which we know there are no individuals who can do this independently, and for which there is also no clearly identifiable collective agent. The second duty to “take responsive steps towards ϕ” implies that agents can take independent steps that lead to the performance of action ϕ, meaning individual scientists and engineers or groups of people whose combined activities lead to responsible nanotechnological development. Based on this we propose the following reformulation of Collins second criterion: “there are no individual researchers or group agents, such as professional organizations or authorities, that bear the duty of RRI, or take independent steps that would lead to RRI, or too many agents with such duties default.” The third criterion relies on a counterfactual conditional, deriving a duty to create a collective from the statement that a collective would be able to bear a duty once it exists: “if, at t^1^, [individual agents] A, …, N each took responsive steps towards there being a collective-that can-ϕ, then, at t^2^, that collective would incur a duty to ϕ” (Collins 2012, 244). So, if individual researchers took steps towards forming a collective agent that *can* responsibly develop nanotechnology than that collective agent would be bestowed with a duty to develop responsibly. Collins does not elaborate on the normative basis for this duty; why it would suddenly emerge once the collective exists. We choose to interpret this as a duty derived from the collective’s capacity to act; since a collective would be able to facilitate responsible development of nanotechnology, this collective would bear this obligation. As this may seem a heavy burden, we will discuss this obligation in more detail in the next section. The reformulation of this criterion becomes: “if researchers organized themselves into a collective that can do RRI, then this collective, once established, would have a duty of RRI.”

The fourth criterion sets limits on the burden that may rest on individual agents to organize themselves: “at t^1^, [individual agents] A, …, N are each able to take responsive steps towards there being a collective-that-can-ϕ at a reasonable expected personal and moral cost.” We translated this into: “researchers involved in nanotechnology development are able to take steps towards organizing themselves without considerable personal and moral costs,” limiting the demanding nature of the collectivization duty for individual researchers.

The fifth criterion states that “other individuals will not successfully take responsive steps towards there being a collective that will incur a stronger duty to ϕ.” The key idea here is that an individual’s collectivization duty is contingent upon the activities of other individuals, who, if they collectivized (a) would fulfill the morally desirable activity, and (b) would as a collective have a stronger duty to ϕ than other collectives. Collins does not specify what constitutes a stronger duty to ϕ for collectives. However, given the action-oriented nature of Collins proposal we assume that collectives with a stronger capacity to ϕ would have a stronger duty to ϕ. Other considerations could also play a role in determining the strength of this collectivization duty. An example is the extent to which the collective has benefited from the situation in which the morally pressing issue emerged. This will be addressed in more detail in the discussion. We propose the following formulation: “there is no reason to believe that other individuals will collectivize into a group that would have a stronger duty to do RRI.”

If these five criteria are met, then this would give individual agents a duty to collectivize, meaning that: “at t^1^, [individual agents] A, …, N each have a duty to take responsive steps towards there being a collective-that-can-ϕ,” which means that individual researchers have to take steps towards organizing themselves into a collective that can develop a mechanism for responsible innovation. Once such a collective is formed, it has a duty to facilitate responsible innovation. As follows from the translation of Collins’ seventh CCD: “at t^2^, once a {A, …, N} collective-that-can-ϕ is formed, that collective has a duty to ϕ.” This is essentially a confirmation of the third criterion in the antecedent. Next: “at t^3^, once the collective has distributed ϕ-related roles, each member with a ϕ-related role has a duty to perform that role” (Collins 2012, 244). Once a collective is formed with the aim of developing responsible innovation methods, and all of the individual researchers understand their roles within this collective, the individual researchers have a duty to perform their individual roles.

This leads to the nanotechnology-adjusted version of Collins proposal for collectivization duties: CCD-Nano:

If:

1. RRI of nanotechnology is morally pressing, and
2. there are no individual researchers or group agents, such as professional organizations or authorities, that bear the duty of RRI, or take independent steps that would lead to RRI, or too many agents with such duties default, and
3. researchers organize themselves into a collective that can do RRI, and this collective, once established, would have a duty to do RRI, and
4. researchers involved in nanotechnology are able to take steps towards organizing themselves without considerable personal and moral costs, and
5. there is no reason to believe that other individuals will collectivize into a group that would have a stronger duty to do RRI

then:

(6) individual researchers have to take steps towards organizing themselves into a collective that can do RRI, and
(7) once this collective is formed, it has a duty to do RRI, and
(8) once this collective has distributed roles to individual researchers, each member has to perform his or her individual roles

This idea of collectivization duties fundamentally shifts the discussion about how to implement RRI in the case of nanotechnology. Rather than focusing on particular harmful impacts and risk impositions that may be difficult to prevent due to limited power or ignorance concerning future developments, it presents something that is within reach of individual researchers: the *duty to organize themselves*. Researchers at all levels in nanotechnology have the individual agency to contribute to this goal, though their activities depend on their position and power within the field (as secured in criterion 4). Individuals know how to create structures and procedures and allocate tasks to create a collective, which can in turn ensure that technological development proceeds in a responsible manner.^3^ Similar work was done during the nascent years of computer science. Individuals in this group collectivized in order to form a new professionalism in their field. They formulated codes of practice and ethical guidelines to describe acceptable ways of practicing software engineering (Gotterbarn 1997). If our argument holds, individuals who take adequate responsive action with a view to creating a collective would be taking an irresponsible moral risk.

## Discussion: Challenges in Applying the Duty to Collectivize in the Nanotechnology Context

Before we can unambiguously apply the criteria for collectivization duties that we modified for the nanotechnology context (CCD-Nano), there are some challenges to overcome. Only then can we use CCD-Nano to establish whether researchers working on nanotechnologies have a duty to collectivize. We will discuss these challenges with a view to improving our approach for future use.

The first objection one could raise to a collectivization duty in nanotechnology is that it is unclear when an issue is morally pressing enough to justify a need for forward-looking responsibility. Held’s example of a person drowning seems morally clear; it is obvious that some sort of action would have to be taken in life or death situations. Something similar can be said for preventing known risks. We can reasonably expect a certain duty to prevent health and environmental risks or negative social impacts. This would, of course, be proportional to the magnitude and likelihood of that particular risk. In the case of uncertain risks, however, this is much more difficult. In the case of nanotechnology, something may have the potential to cause harm due to unforeseen technological developments, but we have no certainty about what exact threats may emerge and no way of calculating probabilities of these events occurring. In such cases a duty to take action is much harder to justify.

Therefore, the key to developing CCD-Nano is to reflect on what would constitute a sufficient reason to expect people to organize themselves. Which concerns warrant collectivization duties and which do not? One way of addressing this issue in the case of nanotechnology could be to take the precautionary principle as an indication of the urgency of the matter. Several scholars have argued in favor of a precautionary approach to uncertain nanotechnological risks (Ahteensu and Sandin 2012; Spruit 2015; Weckert and Moor 2007). This means that when there is reason to expect potential harmful impacts, but solid scientific risk information is lacking, precautions would have to be taken to prevent further harm. If invoking the precautionary principle is seen as a way of establishing the urgency of a matter, this would demonstrate that the nanotechnology field is morally pressing enough to justify a duty to collectivize, or at least to meet criterion 1 of CCD-Nano.

Another issue that emerges is the demarcation of the collection of individuals who have collectivization duties. Who should organize themselves or, in Collins’ terms, who are agents A through N that have to collectivize? We have deliberately avoided talking about ‘nanoengineers’ in this paper as it is quite difficult to demarcate this group. The nanotechnology label is often applied to make research more appealing to funding agencies or publishers, but the individuals using the label do not identify themselves as members of a nanotechnology community. Conversely, some researchers working on nanotechnology eschew the label entirely, believing it to be irrelevant. For example, during the [Anonymized for review] program [Anonymized for review], one faculty speaker at UC Berkeley explicitly did not refer to himself as a nanoengineer, even though he was working at the nanoscale, because he thought it was a useless category. This demonstrates that actors may not feel part of this community at all, even though they may do technically similar work, simply because they label their work differently.

However, the ambiguity of the nanotechnology label is no excuse to avoid responsibility. Researchers in the field of nanotechnology may be what May and Hoffman call a ‘putative group’, a group of people that could organize themselves once they recognize that they share responsibilities (May and Hoffman 1991; Ch. 6). Fortunately, Collins’ proposal is not contingent upon the recognition of a shared identity, but on the recognition of a shared problem. Collins argues in a consequentialist fashion that those people who “*are each able to take responsive steps towards there being a collective*-*that*-*can*-*φ*” (Collins 2012, 244) are our target group, the bearers of collectivization duties. The idea is that those people who can reliably establish an effective group—a group that can reliably perform the morally pressing action—should be expected to organize themselves.

This demarcation based on an efficacy argument is appealing but brings us to a third challenge: how do we know a collective will reliably ensure RRI? Given that it is a key feature of this technological domain that uncertainty and unpredictability impede the construction of adequate risk management strategies, the question remains how one would know beforehand if a collective of researchers can effectively steer technological development and do RRI. The efficacy criterion is underdetermined in this sense. A suggestion could be to add a fairness criterion to the efficacy criterion that is common in consequentialist notions of responsibility, as suggested by Doorn (2012). Many individual scientists and engineers benefit from using the ambiguous ‘nanotechnology’ as a label. It provides them with access to funds and resources. It is not a new idea that such a benefit of membership in a particular group could also give rise to responsibilities towards that group (May and Hoffman 1991). Funding may provide a discerning criterion to distinguish between all individual researchers who *can* collectivize and those who *have a duty* to collectivize because of the benefits they got from the ‘nanotechnology’ label.

## Conclusion

In this paper we established the conditions under which individual researchers have a duty to collectivize in order for research and innovation to be done responsibly. We focused on the field of nanotechnology—specifically on the research setting at UC Berkeley—in order to understand how individual capacity to do RRI is limited, especially in the absence of a collective that can provide a framework for RRI. As an answer to this situation we have proposed a conception of individual responsibility in RRI based on a duty to collectivize. We have explored how this could be a fruitful approach for nanotechnology while acknowledging there are still hurdles to overcome, namely: deciding what concerns provide sufficient grounds to expect people to collectivize, determining the reasons to demarcate groups of individuals who should constitute the collective, and establishing whether a collective can be expected to do RRI in a reliable manner.

We expect our approach to be valuable not only in discussions concerning responsibility in the nanotechnology field. Other emerging and converging research fields will face similar struggles in establishing a professional and ethical identity. Especially in the early days of a new field of science and engineering, the ethical concerns and fears that emerge may have to be dealt with quickly and adequately. Our proposal for the duty of collectivization encourages individuals to build structures that can steer technological development in directions that make a positive contribution to our world, while maintaining a situation in which this new field of research and innovation can flourish.

## Footnotes

1 The absence of a professional nanoengineering identity is confirmed by the absence of a stand-alone nanoengineering undergraduate or doctoral program. The BNNI organizes a designated emphasis in Nanoscale Science and Engineering for PhD students. UC Berkeley defines a designated emphasis as “an area of study constituting a new method of inquiry or an important field of application relevant to two or more existing doctoral degree programs. It is not a free-standing degree program, but must be added as an additional major along with an existing doctoral degree program” (BNNI 2014).

2 Currently, much of the ethical debate about nanotechnologies takes place outside of the nanotechnology community, in applied ethics or social scientific journals, such as the journal NanoEthics. Alongside the development of scientific advances in nanotechnology has come extensive research into the ethical aspects of nanotechnology, specifically in the field of nanoethics, and more generally in STS and engineering ethics. Discussions concerning nanotechnology have ranged from debates on practical considerations related to chemical hygiene to dystopian scenes of world destruction (Drexler 1986; Gordijn 2005; Lin and Allhof 2007). However, many of the findings that SS shared from her field of ethical inquiry were novel to DG and DR.

3 The result of this may be alliances with people external to the research field (like the alliance between the authors of this paper), such as nanoethicists who help analyze what responsible development of nanotechnologies actually entails.
